# Effectiveness and cost-effectiveness of a transdiagnostic intervention targeting alcohol misuse and psychological distress for men in Ukraine: study protocol for a randomised controlled trial

**DOI:** 10.1186/s13063-025-09143-8

**Published:** 2025-10-09

**Authors:** Catharina F. van der Boor, Vita Kachai, Kateryna Harbar, Alona Pastukhova, Melissa Neuman, Helen A. Weiss, Giulia Greco, Carl May, Abhijit Nadkarni, Bayard Roberts, Sergiy Bogdanov, Daniela C. Fuhr

**Affiliations:** 1 Faculty of Public Health and Policy, London School of Hygiene and Tropical Medicine, 15-17 Tavistock Place, London, WC1H 9SH UK; 2 Centre for Mental Health and Psychosocial Support, National University of Kyiv-Mohyla Academy, 2 Skovorody St., Kiev, 04070 Ukraine; 3 MRC International Statistics and Epidemiology Group, Faculty of Epidemiology and Population Health, London School of Hygiene and Tropical Medicine, Keppel Street, London, WC1E 7HT UK; 4 Centre for Global Mental Health (CGMH), Department of Population Health, London School of Hygiene & Tropical Medicine, London, UK; 5 Addictions and Related Research Group, Sangath, Goa India; 6 Department of Prevention and Evaluation, Leibniz Institute for Prevention Research and Epidemiology, Achterstraße 30, 28359 Bremen, Germany; 7 Health Sciences, University of Bremen, Bremen, Germany

**Keywords:** Conflict, Alcohol, Mental distress, Scalable interventions, Randomised controlled trial

## Abstract

**Background:**

Ukraine has experienced armed conflict since 2014, with significant escalation in 2022. Since then, an estimated 3.7 million people have been internally displaced. Alcohol misuse remains a substantial public health challenge in Ukraine, with high levels of psychological distress among the displaced population. The current study aims to evaluate the effectiveness and cost-effectiveness of a transdiagnostic intervention (CHANGE) to address alcohol misuse and psychological distress through problem-solving therapy and selected behavioural strategies for managing alcohol misuse. We hypothesize that CHANGE, together with enhanced usual care (EUC), will be more effective in increasing the percentage of days abstinent (PDA) than EUC alone.

**Methods:**

This study is a parallel-arm, single-blind, individually randomised controlled trial across Ukraine government-controlled territories. Following informed consent, we will recruit 500 adult war-affected men, randomised 1:1 to EUC and CHANGE, or EUC alone. Inclusion criteria include elevated levels of alcohol use (between 8 and 19, inclusive, on the Alcohol Use Disorder Identification Test); psychological distress (≥ 16 on the Kessler Psychological Distress Scale) and ability to speak Ukrainian or Russian. CHANGE will be delivered over 6 weeks by 14 community-based facilitators, with outcomes assessed at 3 months post-randomisation. The primary outcome for CHANGE is the PDA from alcohol at 3 months, measured using the Timeline Follow Back. Secondary outcomes include percentage days of heavy drinking, alcohol misuse, psychological distress (depression, anxiety, and posttraumatic stress disorder), functional disability, intimate partner violence perpetration and health economics indicators at 3 months. The primary analysis will follow an intention-to-treat approach. A mixed-methods process evaluation will examine facilitator competency, recruitment, retention/completion, appropriateness, dose received, fidelity and feasibility of delivery and acceptability.

**Discussion:**

CHANGE is the first intervention aiming to address alcohol misuse and psychological distress in an active conflict setting.

**Trial registration:**

ISRCTN14881856. Registered on 5th of July 2024.

**Supplementary Information:**

The online version contains supplementary material available at 10.1186/s13063-025-09143-8.

## Administrative information

Note: the numbers in curly brackets in this protocol refer to SPIRIT checklist item numbers. The order of the items has been modified to group similar items (see http://www.equator-network.org/reporting-guidelines/spirit-2013-statement-defining-standard-protocol-items-for-clinical-trials/).

**Table:** 

Title {1}	Study Protocol: Investigating the Effectiveness and Cost-effectiveness of a Transdiagnostic Intervention Targeting Alcohol Misuse and Psychological Distress for Men in Ukraine through a Randomised Controlled Trial.
Trial registration {2a and 2b}.	Registration was done on the 5th of July 2024 with the International Standard Randomised Controlled Trial Number (ISRCTN) (Ref: ISRCTN14881856). It was approved by the London School of Hygiene and Tropical Medicine ethics committee on the 17th of April, 2024 (Ref: 30,122), and by the National University of “Kyiv-Mohyla Academy” ethics committee on the 3rd of June, 2024 (Ref: FWA00030125).
Protocol version {2}	Version 4, 15/07/2024
Funding {4}	This study has been funded by the NIHR–Wellcome Partnership for Global Health Research (Ref: HSRP496). The funding body has had no role in the design or submission of this protocol. Furthermore, they will have no role in the collection, management, analysis, and interpretation of data.
Author details {5a}	Catharina F. van der Boor^1^, Vita Kachai^2^, Kateryna Harbar^2^, Alona Pastukhova^2^, Melissa Neuman^3^, Helen A. Weiss^3^, Giulia Greco^1^, Carl May^1^, Abhijit Nadkarni^4,5^, Bayard Roberts^1^, Sergiy Bogdanov^2^, Daniela C. Fuhr^1,6,7^ ^1^Faculty of Public Health and Policy, London School of Hygiene and Tropical Medicine, 15–17 Tavistock Place, London, WC1H 9SH, UK ^2^Centre for Mental Health and Psychosocial Support, National University of Kyiv-Mohyla Academy, 2 Skovorody St., Kyiv 04070, Ukraine ^3^MRC International Statistics and Epidemiology Group, Faculty of Epidemiology and Population Health, London School of Hygiene and Tropical Medicine, Keppel Street, London, WC1E 7HT, UK ^4^Centre for Global Mental Health (CGMH), Department of Population Health, London School of Hygiene & Tropical Medicine, UK ^5^Addictions and Related Research Group, Sangath, Goa, India ^6^Department of Prevention and Evaluation, Leibniz Institute for Prevention Research and Epidemiology, Achterstraße 30 D-28359, Bremen, Germany ^7^Health Sciences, University of Bremen, Germany
Name and contact information for the trial sponsor {5b}	This study is sponsored by the London School of Hygiene and Tropical Medicine (Ref:2024-KEP-1050). RGIO@lshtm.ac.uk London School of Hygiene & Tropical Medicine Keppel Street, London WC1E 7HT
Role of sponsor {5c}	The sponsor has had no role in the design or submission of this protocol. Furthermore, they will have no role in the collection, management, analysis, and interpretation of data.

## Introduction

### Background and rationale {6a}

The conflict between Russia and Ukraine began in 2014 and escalated into a full-scale invasion on February 24, 2022. Since 2022, approximately 3.7 million people have been internally displaced, 6.5 million Ukrainian refugees have been recorded globally and an estimated 14.6 million people are in need of humanitarian assistance [1].

The World Health Organisation (WHO) estimates that 9.6 million people in Ukraine are currently at risk of or living with mental health conditions [2]. The Russian invasion of 2014 [3, 4] and the 2022 escalation have caused significant psychological harm to the Ukrainian population [5]. A 2016 survey reported that 21% of respondents had post-traumatic stress disorder (PTSD) [3]. Another survey conducted in the same year found high prevalence rates of PTSD (32%), depression (22%), and anxiety (17%) amongst internally displaced persons in Ukraine [6]. Mental health worsened in Ukraine following the 2022 invasion, with the most notable increases being in anxiety, depression, and loneliness [7]. A nationwide study conducted in 2022 looking at trauma exposure due to war-related stressors as a result of the most recent invasion found that all participants had been exposed to at least one war-related stressor, with 26% experiencing PTSD and 15% suffering from complex PTSD [5].

Mental health conditions, particularly depression and anxiety, have a strong comorbidity with alcohol misuse [8]. Adversity, including lack of a supportive environment, demoralization, mental health symptoms and the normalization of alcohol misuse all contribute to high prevalence rates of alcohol use disorders (AUDs) in Ukraine [9, 10]. Over 10,000 deaths in Ukraine were related to alcohol consumption in 2019, with more than 80% of these deaths occurring among men [11]. Similarly, a WHO survey conducted over two weeks at the end of November in 2023 in Ukraine revealed that 77% of respondents were current drinkers, of whom almost half (49.2%) reported heavy episodic drinking over the last 12 months. Among those who engaged in heavy episodic drinking, 64% were men [12]. However, the report noted that 22% of respondents reported a decrease in alcohol consumption since the full-scale invasion, which can be partly attributed to government-imposed restrictions on alcohol sales during the initial phases of the conflict, which were revoked after 3 months. In comparison, 6% reported they had started drinking more, and 3% returned to drinking after a period of abstinence [12].

Responding to both AUDs and psychological distress can help improve outcomes for both conditions; however, interventions that are freely available and easy to scale up are lacking. Ukraine’s mental healthcare system is characterized by a high priority for specialized and/or institutionalized settings, is highly fragmented [13], and provides limited options for community-based mental health services [14]. Despite a significant demand for mental healthcare and support, an estimated 74% of internally displaced persons (IDPs) in Ukraine are not accessing mental health services [6]. Barriers to help-seeking include a preference for self-medication, cost of health care, mental health stigma, lack of information, healthcare providers’ poor understanding of mental healthcare and the perceived poor quality of services [6]. The accessibility of healthcare services has in general significantly deteriorated due to the escalation of war, and Ukrainians face major barriers to accessing care [15]. Further, there mental health stigma is common among individuals and healthcare providers [13, 16], particularly for individuals with AUDs [13]. Due to this large treatment gap for mental health disorders in Ukraine, there is an urgent need to evaluate effective low-intensity interventions delivered by non-specialists (task-sharing) available to meet current and future mental health needs [17].

In response to the large global health burden for mental disorders, the WHO developed Problem Management Plus (PM +), a brief, low-intensity community-based psychological intervention delivered by lay healthcare workers [18]. PM + consists of five sessions and was designed for adults experiencing symptoms of common mental disorders such as depression, anxiety, stress or grief, alongside self-identified practical problems. As a transdiagnostic intervention, PM + uses common principles across various mental disorders without being tailored to a specific diagnosis. Research has shown that PM + effectively reduces stress and promotes positive mental health outcomes [19, 20]. The CHANGE intervention was developed through a systematic intervention development process [21] is informed by PM + , and incorporates additional evidence-based strategies to address alcohol misuse, which were identified through a meta-review.

This paper presents the trial protocol to test the effectiveness and cost-effectiveness of ‘CHANGE’, among war-affected men in Ukraine. This trial is part of a broader five-year project aimed at addressing alcohol misuse and related mental health comorbidities (i.e. depression, anxiety and post-traumatic stress symptoms) in war-affected men in Uganda and Ukraine [22]. We previously published the protocol for the comparison trial evaluating the effectiveness and cost-effectiveness of CHANGE in Uganda [23].

### Objectives {7}

The objective of this study is to assess the effectiveness and cost-effectiveness of the CHANGE intervention. Our primary hypothesis is that the combination of the CHANGE intervention and enhanced usual care (EUC) will be more effective than EUC alone in increasing percentage of days abstinent (PDA) at the 3-month follow-up, which is our primary outcome. Secondary hypotheses are that CHANGE combined with EUC will outperform EUC alone in reducing psychological distress, symptoms of depression, anxiety and PTSD; levels of disability; and intimate partner violence at the 3-month follow-up assessment. We also hypothesize that CHANGE combined with EUC will be more cost-effective in terms of cost per DALY averted and/or QALY gained, and result in greater cost savings for the user and the provider compared to EUC alone.

### Trial design {8}

The current study uses a parallel-arm, superiority, single-blind and individually randomised controlled trial (RCT) design with equal allocation between the control and interventions arms. CHANGE will be fully delivered in a remote format (online or via telephone). A mixed-methods process evaluation will be carried out investigating competency of facilitators delivering the intervention, dose received, fidelity and feasibility of intervention delivery and acceptability of participants with regards to CHANGE. The economic evaluation will investigate the cost-effectiveness of CHANGE compared to EUC from a user and provider perspective.

## Methods: participants, interventions and outcomes

### Study setting {9}

The CHANGE intervention will be implemented in a remote format (online via a voice or video call with a facilitator) among war-affected Ukrainian men living across all the Ukraine government-controlled territories. There are currently an estimated 3.7 million IDPs in the government-controlled territories (of whom 41% are male) [24], as well as an estimated 1.2 million veterans returning from military service [25]. It was necessary to focus solely on Ukraine government-controlled territories to mitigate against potential physical and psychological risks for participants residing in territories controlled by the Russian military and Crimea. We decided to implement the intervention in a fully remote format following participant preferences in the feasibility trial. The feasibility trial was conducted between September 2023 and April 2024 in Kyiv and Dnipro, in person and online (unpublished data). In the feasibility trial, martial law and intensified military recruitment negatively impacted on the recruitment and willingness of men to attend in-person activities due to fears of conscription and being seen in public. Concerns about personal data collection amid ongoing military recruitment, the changing safety situation, and attending sessions in person, also affected participant retention in the face-to-face format. Consequently, for the effectiveness trial the decision was made to conduct the program exclusively in a remote format.

### Eligibility criteria {10}

Study participant inclusion criteria are Ukrainian adult men (age ≥ 18 years) residing in a territory controlled by the Ukrainian government with (i) a score between 8 and 19 (inclusive) on the Alcohol Use Disorder Identification Test (AUDIT) [26], indicating alcohol misuse; and (ii) a score equal to or greater than 16 on the Kessler Psychological Distress Scale (K10) [27], indicating high levels of psychological distress. Finally, participants must be able to speak Ukrainian and/or Russian spoken in Ukraine.

Exclusion criteria are (i) men with severe AUD (AUDIT score ≥ 20) or non-hazardous alcohol consumption (AUDIT < 8 [26]; (ii) men identified as being at imminent risk of suicide or other life-threatening risk (determined through three brief questions: ‘In the last month, have you had thoughts about suicide (or harming another person) or a suicide plan (or a plan to harm another person)?’; ‘Do you have ways to carry out this plan?’; and ‘Have you tried to kill yourself (or to kill or harm another person) in the past month?’); (iii) men who express high levels of homicidal ideation (determined by the person having thoughts, plans, ways to carry out the plan and/or experience in harming another person). These individuals will be offered a referral to a psychiatrist; (iv) men identified as having signs of severe mental disorders such as psychosis and/or severe cognitive impairment, assessed through a checklist of observable signs (e.g. not understanding questions, presenting confused speech, or limited communication skills); (v) men who have received other formalized brief psychological interventions (e.g. PM + , Common Elements Treatment Approach (CETA)) in the last year, as well as those who have received treatment for substance misuse (e.g. Alcoholics Anonymous, detoxification, replacement therapy programs) at any point in their lifetime; and (vi) men who are actively involved in military action and those who reside outside of Ukraine will also be excluded. Outcome assessors will carry out the screening procedures and will be trained on providing support for referral pathways to specialised care.

Whilst the current study focuses on men, if women with harmful alcohol use and elevated psychological distress approach the study, they will be offered the option to complete the consent procedures and screening. If eligible, they will be offered the outcome measures, the CHANGE intervention and EUC. However, their data will not be used in this trial and will be analysed separately.

Fourteen community-based providers, referred to as ‘facilitators’, have been recruited and trained to deliver CHANGE in Ukraine in a remote format. Inclusion criteria for the facilitators are (1) preferred experience working with men who are veterans, internally displaced persons (IDPs), and other population groups affected by the war, (2) preferred prior experience in the social sector or in the field of humanitarian response, (3) speak Ukrainian and (4) have stable access to internet. Recruited facilitators included: psychologists by training (*n* = 10), psychologists in training (*n* = 2), social workers (*n* = 1), providers without mental health background (*n* = 1) with relevant experience in psychosocial activities and/or community work. Facilitators are based in different regions across Ukraine including Kyiv (*n* = 4), Dnipro (*n* = 3), Lviv (*n* = 1), Ternopil (*n* = 1), Khmelnytskyi (*n* = 1), Vinnytsia (*n* = 1), Kharkiv (*n* = 1) and Poltava (*n* = 2). Lastly, facilitators work across different organizations including the National University of Kyiv-Mohyla Academy (NaUKMA) Mental Health Centre, NGOs, community-based organizations, governmental organizations, and private practice.

### Who will take informed consent? {26a}

Verbal informed consent will be documented digitally by the outcome assessors before the screening and baseline assessment. For this, participants will receive a participant information sheet and consent text via email or online messaging app in advance (depending on their preference). At the beginning of the consent procedure that takes place on the phone or online, the outcome assessor will verbally explain the purpose of the study and answer any questions the participant may have. Participants will provide oral consent to alleviate concerns about being identified for military recruitment, an issue that emerged during the feasibility trial. A similar, yet separate, consent process will be conducted prior to the qualitative interviews for the process evaluation.

### Additional consent provisions for collection and use of participant data and biological specimens {26b}

No biological specimens will be collected during this trial.

## Interventions

### Explanation for the choice of comparators {6b}

Participants in the control arm will be offered EUC in a remote format (online or via the telephone). The participant will be asked to choose to connect via an online platform or to a telephone connection, considering power cuts, personal preference or other technical issues. EUC consists of a consultation session with one of two trained EUC specialists, who will go through an electronic information pamphlet which outlines information on reducing alcohol intake and managing psychological distress, as well as information for participants on alternatively available psychological services they can individually access. EUC was deemed an ethical comparator in the context of war by the research team and ethics committees.

### Intervention description {11a}

In addition to being offered EUC as described above, participants randomised to the intervention arm will be offered six sessions of the CHANGE intervention in a remote format. Based on the feasibility trial, an additional booster session may be included that focuses on reviewing the previously taught strategies (without introducing new ones) that can be provided to participants with high stress levels, providing extra time to practice and internalize them. A convenient online platform for the call will be agreed upon with the participants. These can include, but are not limited to, Zoom, Google Meets, Skype, Telegram or Viber.

The CHANGE intervention is a brief, transdiagnostic psychological intervention, informed by PM + and enhanced through strategies based on empirical evidence, which aim to address alcohol misuse [22, 23]. The initial PM + intervention uses strategies including problem solving, stress management, behavioural activation and accessing social support to address common mental health problems [18]. To develop the CHANGE intervention targeting alcohol misuse, firstly a meta-review was conducted [28] to identify evidence-based strategies for the management of AUDs. After this a systematic intervention adaptation process followed to incorporate strategies into PM + that address alcohol misuse including enhancing motivation to change drinking behaviours. CHANGE is composed of three phases each which include individual weekly sessions of approximately 90 min each with the facilitators (Table 1).

**Table 1:** Outline of the three phases and psychological strategies included in each phase of CHANGE

Phase I	Phase II	Phase III
• Engaging the client • Assessments • Review from the assessments • Information about the CHANGE intervention • Understanding adversity • Understanding alcohol use • Goal setting for drinking • Specifying a change plan	• Changing drinking behaviours • Managing stress • Managing emotions • Managing problems • Behaviour activation • Strengthening social support	• Relapse prevention • Ending the intervention • Optional booster session
Basic helping and motivational skills		

### Criteria for discontinuing or modifying allocated interventions {11b}

If clinical deterioration, an adverse event (AE) or a serious adverse event (SAE) is detected throughout the course of the trial by an outcome assessor or facilitator, it will be promptly reported to the research team, trial sponsor, ethics committees and Data Safety and Management Board (DSMB), see Sect. 22 for further details. Where an AE or SAE occurs, participants will be referred to specialist support if deemed necessary by the research director and research team using previously developed remote referral pathways. Clinical deterioration includes instances where a participant is intoxicated during multiple sessions, reports suicidal ideation, or homicidal ideation. Participants who report suicidal ideation will only be referred to specialist services or withdrawn from the trial if they are at imminent risk of suicide, which involves having a plan in place. Participants referred to specialist support during baseline outcome assessment or the CHANGE intervention sessions will be withdrawn from the trial but may continue to receive the remaining CHANGE sessions. If participants are referred to specialist support at the 3-month outcome assessment, they will not be withdrawn from the trial. All facilitators and outcome assessors have received training in safety protocols for remote service delivery and to ensure adherence to best practice.

### Strategies to improve adherence to interventions {11c}

We recruited 4 licensed mental health care professionals (e.g. clinical psychologists, psychiatrists, experienced counsellors) who received a training-of-trainers program with the aim of carrying out supervision for CHANGE facilitators. These trainers/supervisors then delivered a 6-day in-person training, and 1-day online refresher training to the first cohort of facilitators ((*N* = 13, prior to the start of the trial), and a 7-day in-person training to the second cohort of facilitators (*N* = 9). The training program included education on common mental disorders, basic counseling skills, delivery of the CHANGE sessions and self-care. After the training, 16 facilitators were selected for the trial based on their skills, availability and/or geographical location. Two facilitators decided to discontinue their involvement, leaving 14 facilitators for the trial.

During the upcoming trial, the 14 CHANGE facilitators will be divided into two groups, with two supervisors being assigned to each group for online supervision (approximately 2 h per week per group). The supervisors will rotate on a weekly basis within their respective groups to ensure that each supervisor conducts two group supervisions per month, resulting in a weekly group-supervision opportunity for each facilitator. A gradual decrease in frequency of group supervisions is planned throughout the duration of the trial, dependent on the facilitators’ ongoing clinical competencies, decision-making skills, and adherence to protocols.

To carry out fidelity checks, participant sessions for fidelity assessments will be randomly selected by the statistician using Stata 18.0 (College Station, TX, USA) prior to the start of recruitment. An equal number of recordings will be randomly selected across all six sessions, with each participant recorded only once. The M&E lead tracks selected sessions for fidelity recordings on a weekly basis. During consent, participants explicitly give permission for session recordings for quality assurance. Fidelity checklists have been developed based on the strategies and components covered in each of the CHANGE sessions. Fidelity assessments will be completed by the trainers/supervisors.

### Relevant concomitant care permitted or prohibited during the trial {11d}

Concomitant care provided by anyone outside the trial team will be permitted. This may include for participants who are identified as being a perpetrator or victim of intimate partner violence at any time during the trial. These participants will be given additional support via online referral pathways to protection services. Wherever possible, we will document referrals to other forms of care/interventions to allow for sensitivity analysis if needed.

Given the ongoing conflict in Ukraine, all participants will be asked to agree with the safety procedures that are in place before meeting with an outcome assessor, EUC specialist, or facilitator. Including that in the case of an air raid siren taking place in either the CHANGE staff or participants’ location, the online meeting will be stopped, and both participants and the CHANGE staff will prioritise seeking safety. Safety will be sought following local safety directions.

### Provisions for Posttrial care {30}

The 14 facilitators trained to deliver the CHANGE intervention in Ukraine will continue their work and provide care to the community after the trial concludes. Additionally, CETA, which is implemented by the community-based NGO WordsHelp will be available for participants who need additional care. CETA is a treatment program for adults presenting with transdiagnostic problems (e.g. PTSD, depression, anxiety and substance use) developed specifically for use with community workers in low- and middle-income countries and has been proven effective in the context of Ukraine [29]. Participants may be referred to or choose to self-refer to these services. Where participants access CETA during the trial they will be excluded from the analysis.

### Outcomes {12}

The outcome assessors will conduct online data collection using tablets and the electronic data capture system ‘Open Data Kit’ (ODK, https://getodk.org) in a remote format (online, via Zoom, Google Meets, Viber, Telegram, other platforms or via the telephone). Outcome assessment takes approximately 2 h. The outcome assessors, EUC specialists and phone operators each received a separate 3-day online training. During the training, outcome assessors focused on using the outcome measures in ODK, general interviewing skills (including role play exercises), learnt about standard operating procedures, and safety procedures. Two of the outcome assessors were previously involved in the feasibility trial for CHANGE. The EUC specialists and telephone operators each received training in their own SOPs and research ethics. All data collectors receive regular supervision from the field team lead, and none are involved in delivering the intervention.

**Fig. 1:**
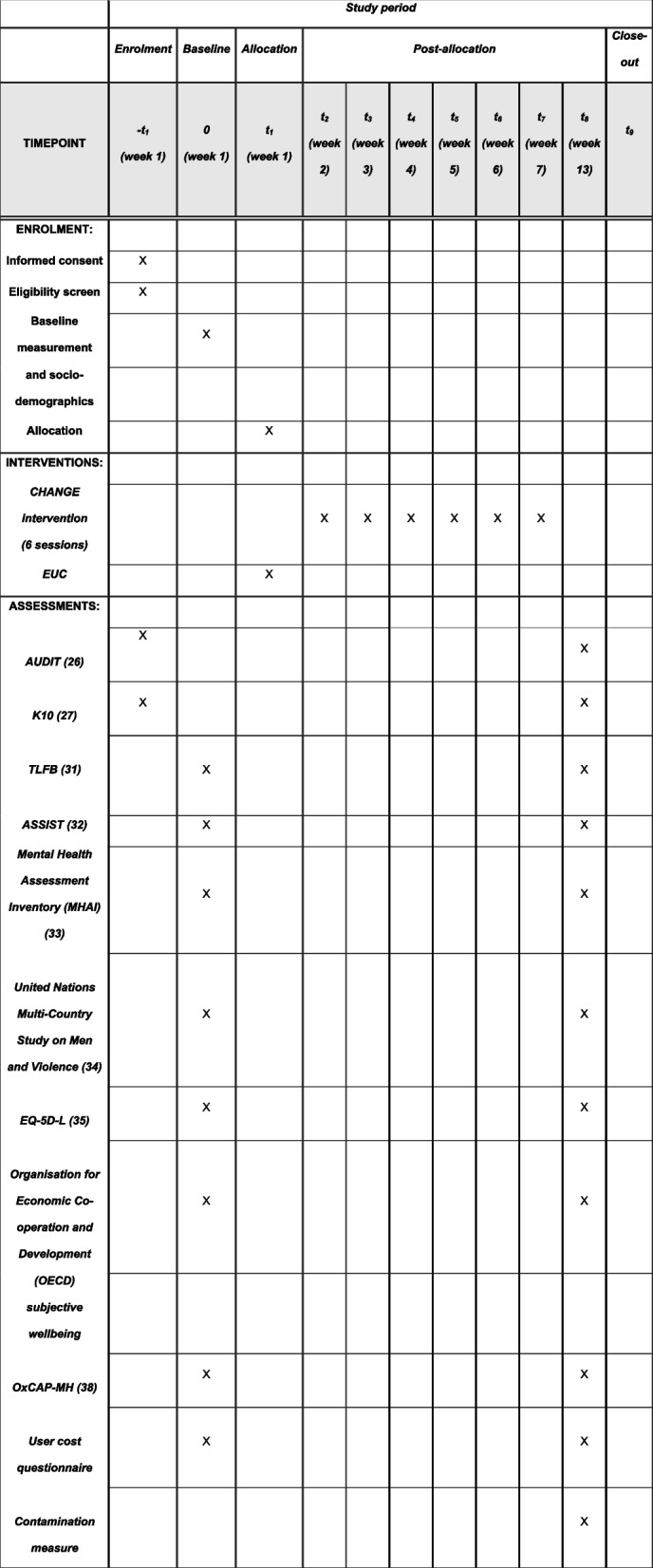
SPIRIT figure including planned enrolment, allocation, intervention delivery and outcome assessments for the CHANGE intervention (adapted from Van Der Boor et al., 2024 [23])

Outcome assessments will take place during baseline, and 3-months post-randomisation. Similar to the trial in Uganda [23], the primary outcome is the percentage of days abstinent (PDA) at the 3 months’ outcome assessment, adjusted for baseline PDA. Secondary outcomes will include the 3-months assessment in percentage days of heavy drinking, alcohol misuse, psychological distress, depression, anxiety, PTSD, functional disability and perpetration of intimate partner violence. Additionally, quality of life, subjective wellbeing and capabilities-based wellbeing will be measured as secondary outcomes. The outcomes of the trial are summarised in Table 2, with a description of the assessment measures of the outcomes in Table 3. Furthermore, sociodemographic data will be collected during the baseline assessment including level of education, socio-economic status, data on whether they have been displaced and their involvement in military action. At follow-up, we will also collect data on socio-economic status to identify changes. A contamination measure will be used at the 3-month follow up to measure potential contamination of intervention strategies between the two intervention arms. Table 4 describes the socio-demographic data collected at baseline, and Fig. 1 provides an overview of the planned enrolment, allocation, intervention delivery and outcome assessments.

**Table 2:** Primary and secondary outcomes of the trial (table adapted from Van Der Boor et al., 2024 [23])

	Source of data (see Table 3 for details of each measure)	
**Primary outcome**	Measure	End point (3 months follow-up)
Percentage of days abstinent	Timeline Followback (TLFB) [31]	X
**Secondary outcomes**		
Percentage days of heavy drinking	TLFB	X
Alcohol Misuse	• Alcohol Use Disorders Identification Test (AUDIT) [26] • Alcohol, Smoking and Substance Involvement Screening Test (ASSIST) [32]	X
Psychological distress	Kessler-10 (K10) [27]	X
Depression	Mental Health Assessment Inventory (MHAI), depression sub-scale [33]	X
Anxiety	Mental Health Assessment Inventory (MHAI), anxiety sub-scale [33]	X
PTSD	Mental Health Assessment Inventory (MHAI), PTSD sub-scale [33]	X
Functional disability	Mental Health Assessment Inventory (MHAI) [33]	X
Perpetration of intimate partner violence	United Nations Multi-Country Study on Men and Violence [34]	X
Health economics indicators	• EuroQol 5 dimensions 5 Levels (EQ-5D-5L) [42] • Subjective wellbeing [35] • Oxford Capabilities Mental Health questionnaire (OxCAP-MH) [36] • User cost questionnaire	X

**Table 3:** Details on CHANGE Intervention Trial Outcome Measures (table adapted from Van Der Boor et al., 2024 [23])

Instrument	Description	Outcome	Contextual validity
Timeline Followback	Diary method to retrospectively assess daily estimates of drinking	Percentage days abstinent (PDA): Proportion of past 14 days on which participant was abstinent; analysis based on distribution. It also allows measurement of percentage of days of heavy drinking	Validated for alcohol use assessment including in Ukraine [43]
AUDIT	Ten-item questionnaire to measure alcohol intake, identify potential dependence on alcohol; and experiences of alcohol-related harm. Each item assessed on a scale of 0 to 4	Mean score, and dichotomous (medium vs. high risk) as secondary outcome	Validated for the current project with Ukrainian and Russian speaking men living in Ukraine, paper forthcoming
ASSIST	Eight-item screening questionnaire on estimates of alcohol use, tobacco products, and other drugs across the lifetime and in the past three months. Measured on a scale of 0 to 7	Mean score	Validated in a multisite international study covering Australia, Brazil, India, Thailand, United Kingdom, USA, and Zimbabwe [44]
K10	Ten-item scale on non-specific psychological distress, scored on a scale of 1 to 5	Mean score	Validated in Ukraine including amongst war-affected Ukrainians [45, 46]
MHAI	Thirty-two item scale on depression, post-traumatic stress, anxiety, functioning and alcohol use	Mean score	Locally validated measure with good psychometric properties [33]
United Nations Multi Country Study on Men and Violence	Eleven items are used to measure the prevalence of perpetration of violence in the last 3 months. The items are assessed on a scale of 1 to 4, or 1 to 7	Prevalence of IPV split by type of intimate partner violence (physical, sexual)	Previously validated in Bangladesh, China, Cambodia, Indonesia, Sri Lanka, and Papua New Guinea [34]
EQ-5D-L	Five item questionnaire for measuring health related quality of life across five dimensions: mobility, self-care, usual activities, pain/discomfort, and anxiety/depression, rated on the day. A five-level response option is used	Locally adjusted scoring algorithm	Validated across different contexts including Ukraine (https://euroqol.org/register/obtain-eq-5d/available-versions/)
Subjective Wellbeing	Five items on subjective wellbeing, measuring overall life satisfaction, whether the things the respondent does are worthwhile, and affect; scored on a scale of 1 to 5	Locally adjusted scoring algorithm	Used across countries in the Gallup world poll which covers more than 150 countries, including Ukraine (https://www.gallup.com/analytics/349487/world-happiness-report.aspx)
OxCAP-MH	Sixteen item index on wellbeing, covering various domains of individual well-being including overall health, enjoying social and recreational activities, friendship, and support, having suitable accommodation etc. Scored on a scale of 1 to 5	Mean score	Has been contextually translated and validated by the current research team in Ukraine into Ukrainian and Russian spoken in Ukraine (publication forthcoming)
User Cost Questionnaire	Fifteen item measure on household expenditure specifically designed for this trial to measure the opportunity cost of participating in the CHANGE intervention	The opportunity cost of participating in the CHANGE intervention	Specifically designed and piloted for this trial. Based on the iMTA productivity loss of cost questionnaire

**Table 4:** Sociodemographic data collection

Construct	Measure
Education	Education (no school, primary, secondary, tertiary education, etc.)
Age	In years
Socio-economic status	Self-perceived relative socio-economic status (e.g., income level as multiple-choice question)
Trauma exposure	Life Events Checklist
Use of local substances (e.g., khat, tobacco, marijuana, opioids, synthetic drugs etc.)	Measured developed for study or standardized measure (e.g., ASSIST)
Displacement status	Multiple-choice answer
Length of time displaced	Open-ended answer
Frequency of movement	Open-ended answer
Veteran status	Open-ended answer
Length of military duty	Open-ended answer
Frequency of military duties	Open-ended answer
Marital status	Multiple-choice answer
Having kids	Multiple choice answer
Number of Kids	Multiple choice answer
Separation from wife/kids due to displacement	Multiple-choice answer
Geographical location	Multiple-choice answer
Occupation/work status	Multiple-choice answer
Use of psychotropic medications (e.g., antipsychotics, antidepressants)	Multiple-choice answer

The Enhancing Assessment of Common Therapeutic factors (ENACT) tool [30] will be used to measure the competency of the facilitators to deliver CHANGE through the Ensuring Quality in Psychological Support (EQUIP) platform. These competency assessments were carried out before the start of the trial with 2 cohorts of facilitators by 3 trainers/supervisors who completed their training in rating and using the EQUIP system. The first assessment with the first cohort of facilitators was conducted 2 months after training completion. Facilitators were asked to perform the intervention in pairs and have them recorded independently (via ZOOM) and uploaded to SharePoint. Similarly, we used the same approach with the second assessment for the first cohort, completed before the RCT began. With the second cohort of facilitators, extra time was allocated during the training for the competency assessment activity in person while all three raters were present to conduct the assessment.

We will conduct a mixed-methods process evaluation during the trial, including data collection on competency of facilitators, fidelity and feasibility of delivery, appropriateness, dose, recruitment, retention/completion and acceptability. A nested qualitative study will be carried out with semi-structured interviews taking place at 3-months post-randomisation to further understand the implementation process. Interviews will be done with supervisors (*N* = 4), facilitators (*N* = 14), outcome assessors (*N* = 2), phone operators (*N* = 2), participants in the control arm (*N* = 20), participants in the intervention arm (*N* = 20), family members of participants (*N* = 20) and as many participants as possible who dropped out from the intervention group. Topic guides will be used to explore the processes which will include themes on engagement with CHANGE, recruitment and attendance, program delivery, perceived effectiveness and helpfulness, acceptability of CHANGE for participants and facilitators, feasibility of delivery, as well as opportunity costs in attending CHANGE.

### Participant timeline {13}

Community-based recruitment will be carried out with the help of the implementing partner and NGO WordsHelp who will recruit within their networks and member base using digital flyers and dissemination of information on Facebook and Instagram pages. Furthermore, recruitment will be facilitated online using social media platforms including Facebook, Telegram, Instagram and others by making available a flyer (the advertisement of the trial with the contact details (online registration form or phone number), and through mass media activities. When participants get in touch with the research team, interested participants will be contacted via the telephone by the research assistants responsible for the initial contacting (phone operators). The phone operators will establish whether the participant has a good understanding of the study, collect additional information to see if the person can benefit from participation, check the technological means to participate (e.g. having a stable internet connection, owning a laptop tablet or phone), their platform of preference (e.g. Google Meets, Zoom, telephone) and a date and time to meet with the data collector for eligibility screening. After the initial contacting, the phone operators will send a digital copy of an information sheet and the text of consent form to participants via messaging app or email to read before the remote meeting with data collector.

Outcome assessors will meet the participant at the date, time and on the platform of preference previously established. Participants are given a short safety briefing, including an overview of potential safety risks, and instructions on what to do in high-risk situations (e.g. missile attacks or air raids during the session), and are given the opportunity to ask questions. Participants are asked to give verbal informed consent before the eligibility screening is conducted. Written consent is not requested given the security concerns of signing documents. If verbal consent is provided, the outcome assessors go through the screening tool, and sociodemographic questions with the participant taking note of their responses using ODK. If eligibility criteria are met, outcome assessors continue to the baseline assessment.

Following the baseline assessment, an EUC specialist will join the call or set up a separate call with participant (on a separate day) to go through an electronic information pamphlet (detailing information on reducing alcohol intake and managing psychological distress as well as outlining ways of contacting alternative psychological services). The EUC specialist will also carry out the randomisation procedure, as detailed in the section below. Those randomised to the intervention arm will receive their first CHANGE session the week after (see Fig. 1).

**Fig. 2:**
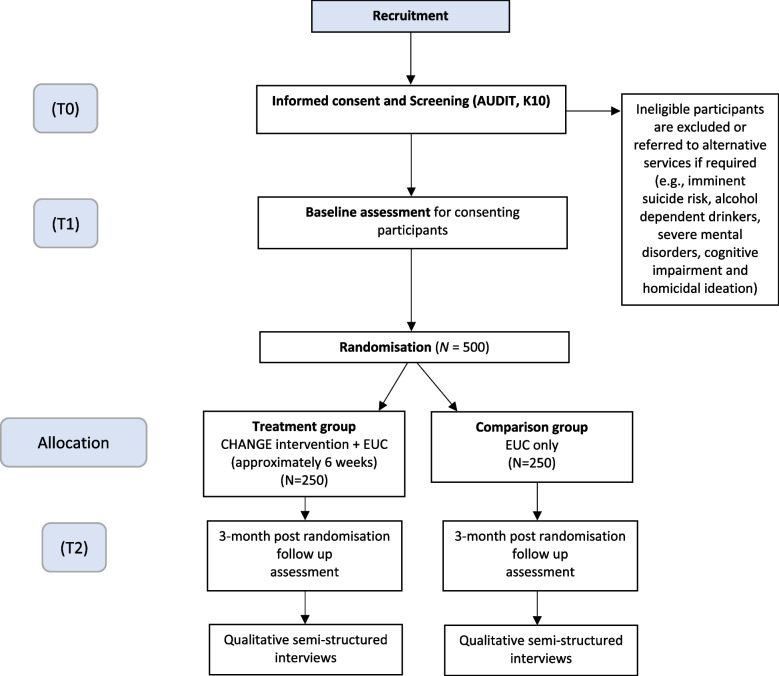
Consolidated standards of reporting trials (CONSORT) diagram (adapted from Van Der Boor et al., 2024 [23])

Participants will be followed up at 3-months after baseline for the outcome assessment. Additional qualitative interviews will be carried out at 3 months follow-up using a purposive sampling strategy. Qualitative interviews will be carried out with all intervention facilitators ( *N* = 14), supervisors (*N* = 4), outcome assessors (*N* = 2) and phone operators (*N* = 2), in addition to family members of participants (*N* = 20), participants in the intervention arm (*N* = 20), participants in the control arm (*N* = 20) and as many participants who dropped out as possible (Fig. 2).

### Sample size {14}

A sample size of 500 enrolled participants (250 in each arm with 1:1 allocation) will provide 90% power (alpha = 0.05) to detect a difference in participants’ PDA from alcohol at follow-up of 55% in the EUC arm vs 68% in the CHANGE arm. This assumes participants’ PDA from alcohol is distributed with a standard deviation of 37% in both arms [37]. Both the effect size and the standard deviation are based on the Counselling for Alcohol Problems (CAP) trial (54% vs. 69%). The sample size calculation accounts for 20% loss to follow-up at 3 months and is based on Nadkarni et al. 2017 [37].

### Recruitment {15}

We focused on Ukraine government-controlled territories to mitigate against potential physical and psychological risks for participants residing in territories currently controlled by the Russian military and Crimea. By government-controlled areas, we refer to areas currently under the administrative and military control of the Ukrainian government. Temporarily occupied territories including parts of Donetsk and Luhansk oblasts, Crimea and parts of Zaporizhzhia, Mykolaiv, Kharkiv and Kherson oblasts were excluded. If the status of these or other territories changes during the trial, their inclusion or exclusion will be adjusted according to this definition. Four different recruitment strategies will be used across government-controlled territories in Ukraine. Firstly, we will disseminate information about the trial on various social media platforms, channels and groups (Telegram, Facebook, Instagram) and mass media: broadcast on the radio, posting news about the trial, interviews and other activities, in addition to setting up partnerships with community-based NGOs, veteran unions and government organizations (e.g. social services) to set up referral pathways to CHANGE. Secondly, community recruiters based at WordsHelp and other local community organisations will use their local connections to divulge information on the CHANGE trial for recruitment purposes. Thirdly, healthcare providers trained in the use of the Mental Health Gap Action Programme (mhGAP) and neurologists will be approached to refer patients who present symptoms of alcohol misuse and other comorbidities to the CHANGE trial. Lastly, digital recruitment flyers will be used for dissemination for partner organizations and social media usage (e.g. Facebook, Instagram), which will include an online registration form to the trial study. Snowball sampling will be used throughout the recruitment process to recruit additional participants. Participants will be offered 250 UAH worth of phone credit or local grocery store card per assessment (screening and follow up). They will also receive the same amount for each session attended (for those randomised into the intervention arm), provided at the end of the treatment course. The date of the first recruitment was 4 September 2024.

## Assignment of interventions: allocations

### Sequence generation{16a}

Stata 16.0 statistical software (College Station, TX, USA) will be used to generate a randomisation sequence by an independent statistician who is not part of the trial team. Randomisation will be blocked of 4, 6 or 8 participants in a random order. Participants will be individually randomised 1:1 to both arms of the trial by the EUC specialist following baseline assessment [23].

### Concealment mechanism {16b}

Concealment of allocation will be ensured using sequentially numbered virtual envelopes. Microsoft Lists will be used to store the randomisation sequence, which will only be accessible to the team members that are unmasked throughout the trial. Virtual envelopes will be assigned to the participant in the order that participants are enrolled into the study. The virtual envelope will display the envelope ID and status (locked/unlocked). The EUC specialist will unlock the respective virtual envelope, after which the envelope status automatically updates to unlocked, the date and time of unlocking is recorded and the randomisation code and allocation is displayed. All actions related to unlocking the virtual envelopes will be tracked using version control mechanisms. This mechanism improves procedural accuracy by demonstrating compliance to standard operating procedures, ensuring there are no errors in the randomisation process. All EUC specialists will be trained on the importance of maintaining the randomisation sequence and weekly data monitoring checks will be done to identify irregularities in the allocation sequence in which envelopes are opened.

### Implementation {16c}

The randomisation database on Microsoft Lists will be prepared by the data manager who is unmasked throughout the trial. The EUC specialists will access the virtual envelopes to enrol participants online, as described above. Once the virtual envelope is unlocked, the EUC specialist will read the text displayed inside the envelope and describe the next steps depending on the allocation of the participant. The EUC specialists will also deliver the EUC online to all of those who are enrolled. Given that all procedures will be conducted remotely, we do not expect any accidental unmasking to take place for the outcome assessors, principal investigators (PIs) or others who are masked.

## Assignment of interventions: masking

### Who will be masked? {17a}

The PI (DF), site PI (SB), co-investigators and outcome assessors will be masked to treatment allocation during the trial. The trial statistician will be masked until the primary analysis is complete. Participants, facilitators, EUC specialists, qualitative researchers and the research director will be unmasked. The field team lead and data manager will also be unmasked as the data manager is responsible for developing the virtual envelopes through Microsoft Lists and the field team lead is responsible for the overall research assistant team support and supervision, carrying out routine data checks and monitoring.

### Procedure for unmasking if needed {17b}

There is no planned procedure for intentional unmasking in this trial. To prevent unintentional masking, all teams have been trained on carrying out the masking procedures, including standard operating procedures. If masking becomes compromised during outcome assessment, the outcome assessor will stop the assessment and notify the research director so a new outcome assessor can conduct the rest of the assessment. The primary outcome will be measured early in the outcome assessment to minimise the likelihood of it being compromised. Furthermore, outcome assessors may become unmasked when detecting an AE or SAE. If the participant remains part of the trial, a new outcome assessor will be assigned (see Sect. 22 for further information on management of AEs and SAEs).

## Data collection and management

### Plans for assessment and collection of outcomes {18a}

Quantitative and sociodemographic data for the CHANGE project will be collected by outcome assessors using ODK on password-protected tablets at baseline and at the 3-month follow-up (see Table 2). Each assessor will use an individual, password-protected tablet assigned specifically to them for completing the screening and outcome assessments. Data will be encrypted and uploaded to the ODK servers at the end of each working day. Daily data checks, including the number of records uploaded, will be conducted, and weekly data reports, which include variable summaries and a summary of missing data, will be generated by the field team lead. Participants will be assigned a unique participant ID to ensure consistent identification across all timepoints. Qualitative interviews will be conducted via the participants’ chosen platform (i.e. Zoom, telephone) in their chosen language (Ukrainian or Russian spoken in Ukraine) and audio recorded for transcription.

### Plans to promote participant retention and complete follow-up {18b}

Participants will be monitored throughout the trial by the field team lead using their unique participant IDs generated through Microsoft Lists Enrolment Database stored on NaUKMA SharePoint with limited access to particular team members who are unmasked. If participants miss an outcome assessment or a CHANGE session, up to three follow-up attempts will be made using their preferred method of contact, including Telegram, Viber, WhatsApp or other. Up to three attempts will also be made to follow up with participants who drop out and those who are lost to follow up at the 3-month outcome assessment.

### Data management {19}

Data will be collected through the ODK platform by the outcome assessors, who will fill in the ODK survey (data) whilst they are on the call with the participant. The ODK server is hosted by the London School of Hygiene and Tropical Medicine and encrypts the data that is stored. A unique participant ID is created automatically by Microsoft Lists Enrolment Database, which will be used to track the participant throughout the trial. The participant ID will be linked to the participants’ name through a password protected database stored on Sharepoint. This Sharepoint will only be accessible to those on the CHANGE team who are unmasked throughout the trial. There are standard operating procedures in place for data entry, monitoring and review and all team members will be given training on these components.

To prevent data loss, a copy of the data will be kept on external hard drives at NaUKMA office (managed by the field team lead) and on SharePoint. The folders and files with the research data stored on the hard drive will be secured by passwords and kept in locked cabinets. Weekly data checks will be carried out by the field team lead including review of the order of randomisation, data entry and range checks. If a data error is found, it will be addressed and logged by the field team lead following the data management plan to ensure there is evidence of changes made.

Sharing of the anonymised and encrypted ODK data will take place using the password protected Sharepoint. The data will not be made available through an open data repository due to the safety and conscription concerns of participants noted during the feasibility trial.

The qualitative data will be audio recorded by the researcher who conducts the interview, and transcription will be done by an external company that has appropriate experience and expertise. Once the data has been transcribed and anonymised, the audio recordings will be deleted. Any data to be used for publication and dissemination will be anonymous and participants will be asked to consent to this.

### Confidentiality {27}

Participants will be assigned with a unique participant ID at the time of registration for participation in the trial which will be automatically generated through Microsoft Lists Enrolment Database stored on Sharepoint. All the information provided by the participant will be saved on the encrypted ODK server and transferred to the password protected Sharepoint. The data management and data security will be aligned to the requirements of General Data Protection Regulations (GDPR). All team members will be trained on maintaining the confidentiality of the participants and protection of the qualitative and quantitative data.

### Plans for collection, laboratory evaluation and storage of biological specimens for genetic or molecular analysis in this trial/future use {33}

This trial does not involve collecting biological specimens for storage or evaluation.

## Statistical methods

### Statistical methods for primary and secondary outcomes {20a}

In line with the protocol published by van der Boor et al. [23], a statistical analysis plan will be written by the statistician based at the London School of Hygiene and Tropical Medicine and approved by the Data Safety and Management Board (DSMB) prior to unmasking of the data and analysis. The quantitative data will be analysed using Stata 17.0. An initial analysis will be conducted to compare participants who did and did not provide consent for participation following initial screening. Further descriptive analysis will then be carried out to compare the baseline characteristics of the participants. If substantial baseline imbalances exist in characteristics that are likely to affect outcomes, these will be adjusted a priori to the outcome analyses.

Binary outcomes will be analysed using logistic regression or negative binomial regression, depending on the distribution. Marginal standardization and delta methods [38] will be used to calculate adjusted risk ratios if logistic regression is used. Continuous outcomes will be analysed using linear regression, and adjusted mean differences reported. Before analysis, distributions of all outcome variables will be inspected, and if necessary, transformations or other modelling options will be considered.

All models will be adjusted for baseline characteristics that differ between the arms as determined by the descriptive analysis and the baseline measures. *P* values will not be used to identify differences between arms at baseline, as these are assumed to be at random. Fully adjusted (if appropriate) model results will be presented and reported using CONSORT guidelines [39]. Intention to treat principles will be used for the primary analysis.

### Interim analyses {21b}

No interim analysis of outcomes is planned.

### Methods for additional analyses {20b}

An economic evaluation will be carried out and will be reported following the Consolidated Health Economic Evaluation Reporting Standards [40]. A cost-effectiveness analysis will be conducted in which an estimation will be made for each participant regarding the intervention costs, disability adjusted life years (DALY) averted and quality adjusted life years (QALY) gained. We will estimate the incremental cost-effectiveness ratios, and a cost-effectiveness plan and acceptability curve will be produced to represent uncertainty. A deterministic sensitivity analysis will be carried out on assumptions made with regards to duration of effect and discount rates. The cost-effectiveness evaluation will be done against a pre-determined threshold and will be compared to other available programs that similarly aim to address alcohol misuse and mental health in Ukraine.

Furthermore, the qualitative data will be transcribed and analysed in the language of the interview (i.e. Ukrainian or Russian spoken in Ukraine). A thematic analysis will be done. The analysis will be done in NVivo © QSR International.

### Methods in analysis to handle protocol non-adherence and any statistical methods to handle missing data {20c}

Data related to outcomes and covariates will be examined for any missing entries, and if the proportion of missing data exceeds 10%, multiple imputation methods may be employed. The results will be presented following the CONSORT guidelines [39], and intention to treat principles will guide the primary analysis of the findings.

### Plans to give access to the full protocol, participant level-data and statistical code {31c}

The participant-level dataset will not be shared due to concerns raised during the feasibility trial, including fears of conscription and access to the data by Russian intelligence. Consequently, consent forms for the full trial were updated to state that data would not be made available to third parties.

## Oversight and monitoring

### Composition of the coordinating centre and trial steering committee {5d}

The trial will be supported by the same Trial Steering Committee (TSC) and Trial Management Committee (TMC) as for the comparison Uganda trial [23]. The TSC will meet every 6 months and will be responsible for the overall governance and oversight of the trial ensuring the conduct is aligned with the protocol and relevant regulations. The TSC is composed of experts on alcohol misuse, mental health and psychosocial support, providing mental healthcare in humanitarian settings and epidemiologists with relevant experience in conducting trials in war-affected settings. On the other hand, the TMC will meet bimonthly and is responsible for providing country-specific advice. It is composed of the PI (DF), and the site PI (SB), who will report to the DSMB and funder regarding the progress of the trial. Furthermore, the field coordinator and work package leads will join the TMC meetings to provide updates on progress.

### Composition of the data monitoring committee, its role and reporting structure {21a}

The CHANGE project is regulated by a DSMB who will meet twice a year to discuss this trial and the trial in Uganda [23]. The members of the DSMB are independent from the TMC and TSC, and are responsible for monitoring the progress of the RCT. The DSMB is composed of the Chair who is a clinical subject matter expert, an independent trialist and statistician, and a second independent clinical subject matter expert. They are the only committee that has access to the unmasked data during the ongoing trial. During the DSMB meetings, the committee will be responsible for deciding whether there are any safety issues within the CHANGE project, report to the TSC and recommend on the continuation of the trial, and consider any requests for the release of the trial data. A DSMB charter is available upon request to the authors.

### Adverse event reporting and harms {22}

The possibility of adverse events which are related to the CHANGE intervention is considered low, and protocols have been developed to identify and address different types of adverse events. From previous experience during the COVID-19 pandemic, during which the service delivery transitioned to a remote format, services to war-affected populations were provided remotely [41]. During that time, adverse events were documented, recognizing the heightened distress associated with war experiences. Remote referral pathways were established, including a comprehensive database of remote service providers, with a preference for in-person referrals where feasible, to connect individuals with additional services, notably psychiatric evaluations and prescriptions. In the current trial, these standard operating procedures will be maintained, namely all members of the clinical team have undergone training in safety protocols for remote service delivery, ensuring adherence to best practices. For high-risk cases, regular check-ins will be conducted to monitor and address evolving situations. The responsible for carrying out these check-ins will depend on the nature of the case (e.g. a psychiatrist or a psychologist). In the past, a limitation of remote service delivery was encountered when clients with potential risks declined to provide detailed contact information, which could impede timely engagement with additional services, such as emergency medical assistance. For the current trial, if a client is considered to be at risk of an AE or SAE, we will request consent for having the contact number of a family member or someone from the clients’ personal sphere to mitigate this risk.

A critical incident register will be maintained during the study to record specific SAEs and AEs. An overview of the types of SAEs and AES can be found in Table 5. SAEs and AEs that are detected during any of the outcome assessments or intervention sessions will be disclosed immediately to the local research director and site PI who will become unmasked. For each individual event, a report will be written. Based on the type of event, the participant will be asked to give consent for further evaluation, assessment, and/or follow-up by a designated health professional (see Table 6). If the participant experiencing an AE or SAE was in the intervention arm of the trial, and once they are referred to further care their data will be excluded from the analysis. Nonetheless, if they are able to, they can receive further CHANGE sessions. The exception to this is referrals for intimate partner violence, whereby participants will be encouraged to access protection services, but will not be excluded from the analysis.

**Table 5:** Expected and unexpected SAE and AEs (adapted from Van Der Boor et al., 2024 [23])

	Expected	Unexpected
SAE	Victimization (violence against the trial participant or nuclear family)	Death of the trial participant due to suicide
	IPV (violence against family members by participant, or violence against participant by family member)	Death of the trial participant due to other causes
	Suicide attempt	Hospital admission of trial participant due to a psychiatric problem
	Homicidal ideation	Planning and/or going through with the homicidal ideation
	Stigmatisation	Hospital admission of trial participant due to other causes (i.e. serious medical emergency)
	Serious lack of food / Critically unsatisfied basic needs	
AE	Clinical deterioration of the participant	
	Emotional distress caused by a trial procedure (either by the outcome assessment or the intervention delivery)

**Table 6:** SAE referral according to intervention arm

SAE	Reported at outcome evaluation in both arms, referral to:	Reported by facilitator in the intervention arm only, referral to:
Hospital admission of participant due to serious medical emergency^a^	General physician (GP), family doctor	GP, family doctor
Hospital admission of participant due to a psychiatric problem	Independent psychiatrist, Psychiatrist in the hospital	Independent psychiatrist, Psychiatrist in the hospital
Victimisation (violence against the participant)	Independent psychologist, social worker, humanitarian agency representative	Intervention supervisor
Severe IPV perpetrated by participant (violence against family members by participant) that includes a risk of severe injury and/or death	Independent psychologist, social worker, humanitarian agency representative	Intervention supervisor
Suicide attempt	Independent psychiatrist, Psychiatrist in the hospital	Independent psychiatrist, Psychiatrist in the hospital
Homicidal ideation	Independent psychiatrist, Psychiatrist in the hospital	Independent psychiatrist, Psychiatrist in the hospital
Stigmatization^a^	Intervention supervisor	Intervention supervisor
Death of the trial participant	N/A	N/A
Any violence toward others	Independent psychiatrist, social worker, humanitarian agency representative	Independent psychiatrist,
Serious lack of food/ Critically unsatisfied basic needs	GP, primary health care worker (nurse/ midwife)	GP
**AE**		
Clinical deterioration of trial participant, including a high risk of a suicide attempt	Independent psychiatrist, Psychiatrist in the hospital	Independent psychiatrist, Psychiatrist in the hospital
Emotional distress caused by a trial procedure (either by the outcome assessments or the intervention delivery)	Independent psychologist, Psychiatrist in the hospital	Independent psychologist, Psychiatrist in the hospital

^a^Prior to referral to the nominated healthcare professional, these SAEs will have an independent clinician review the SAE narrative to determine if this is indeed a SAE

During the 3-month follow-up assessment, all participants will be asked to respond to the safety questions (related to suicide and homicidal ideation) again. In the case of an SAE detection, an independent psychiatrist will assess the participant, and the SAE report will be completed. Participants with safety issues identified during the follow-up will not be excluded from the trial. These questions aim to identify any problems and provide timely referral pathways.

When an SAE is related to stigma, a report will be written as described above, and an evaluation will be conducted by an independent healthcare provider to ensure quality and correct identification of these types of SAEs. Irrelevant of the cause, for events determined by the independent clinician to be SAEs, the person who became aware of the event (outcome assessor or facilitator) will then forward the SAE for further referral to the appropriate professional. The time from initial detection of a potential SAE by the research team (e.g. outcome assessors and facilitators), to the responsible team member (outcome assessor or facilitator) sending the SAE/AE report to the nominated health professional is two working days. Following these 2 days, if it classifies as an SAE, the responsible team member will inform the research director, who will prepare and submit the report to the site PI, who will send it to overall PI, DSMB and respective ethics committees within 5 working days of detection. All SAEs and AEs will be followed up until they have abated, or until a stable situation has been reached. AEs will not be reported to the DSMB but will be included in the annual ethics report.

### Frequency and plans for auditing trial conduct {23}

A bi-annual meeting will be held with the TSC to review the progress and ensure that the trial is conducted in accordance with the protocol and relevant regulations. The DSMB will similarly meet biannually with the aim of monitoring the RCT data. The DSMB is the only body involved in CHANGE that has access to the unmasked data during the trial. Furthermore, a yearly report will be submitted to the Ethics Committee at the London School of Hygiene and Tropical Medicine (LSHTM) to detail progress. These three bodies are independent from investigators and the sponsor, and will be overseeing both the CHANGE trial in Ukraine and Uganda.

### Plans for communicating important protocol amendments to relevant parties (e.g. trial participants, ethical committees) {25}

Protocol amendments will only be implemented following approval by the London School of Hygiene and Tropical Medicine ethics committee and local NaUKMA ethical approval.

### Dissemination plans {31a}

An ISRCTN public trial registration has been completed (ISRCTN14881856). The findings of the effectiveness and cost-effectiveness trial, and process evaluation in Ukraine will be submitted for peer-reviewed publication in international journals. The results will also be shared with key stakeholders (e.g. Ministry of Health, heath clusters, non-governmental organisations, community organisations) through individual country reports and briefs in English, Ukrainian and Russian spoken in Ukraine. Other outputs will include presentations at international conferences and workshops, and findings will be circulated amongst the humanitarian community on platform used by humanitarian workers (e.g., MHPSS.net and MHIN). The next steps in the research project will involve examining the scalability of the CHANGE intervention through the health system and other humanitarian sectors in Ukraine.

## Discussion

The current paper details a trial assessing the effectiveness and cost-effectiveness of the CHANGE intervention in reducing alcohol misuse and psychological distress in war-affected men in Ukraine. Following the current research, an open-access manual developed for implementing the CHANGE intervention in Uganda and Ukraine will be made publicly available. Should the CHANGE intervention prove effective, it can be adopted in other war-affected regions to further evaluate its effectiveness and cost-effectiveness in diverse contexts.

Additionally, the process evaluation of the CHANGE intervention’s implementation will provide valuable insights into the delivery of public health interventions amid ongoing conflict, such as the one in Ukraine. This evaluation will examine the impact on intervention delivery, the implementers, and the overall outcomes of the intervention and will facilitate important reflections around the use of implementation research and frameworks in unstable settings.

### Trial status

The current paper is based on the 4th version of the study protocol (date: 06/02/2025). The first recruitment took place on September 4th, 2024 and we expect the final recruitment to be completed in 2026.

## Supplementary Information

Supplementary Material 1.

## Data Availability

The datasets generated during and/or analysed during the current study will not be made publicly available as participants have not consented to this.

## Abbreviations

AE: Adverse events
AUDs: Alcohol use disorders
AUDIT: Alcohol Use Disorder Identification Test
CAP: Counselling for alcohol problems
CETA: Common Elements Treatment Approach
CONSORT: Consolidated Standards of Reporting Trials
DALY: Disability-adjusted life years
DSMB: Data Safety and Management Board
ENACT: Enhancing Assessment of Common Therapeutic factors tool
EUC: Enhanced usual care
EQUIP: Ensuring Quality in Psychological Support platform
EQ-5D-5L: EuroQol 5 Dimensions, 5 Levels
GDPR: General Data Protection Regulations
GP: General physician
IDPs: Internally displaced persons
ISRCTN: International Standard Randomised Controlled Trial Number
ITT: Intention to treat
K10: Kessler Psychological Distress Scale
LSHTM: London School of Hygiene and Tropical Medicine
MHAI: Mental Health Assessment Inventory
mhGAP: Mental Health Gap Action Programme
NIHR: National Institute for Health Research
NaUKMA: National University of Kyiv-Mohyla Academy
OECD: Organisation for Economic Co-operation and Development
ODA: Official Development Assistance
ODK: Open Data Kit
PDA: Percentage of days abstinent
PIs: Principal investigators
PM +: Problem Management Plus
PTSD: Post-traumatic stress disorder
QALY: Quality-adjusted life years
RCT: Randomised controlled trial
SAE: Serious adverse event
TMC: Trial Management Committee
TSC: Trial Steering Committee
WHO: World Health Organisation
